# Supplementary material for: Probing the pathways of free charge generation in organic bulk heterojunction solar cells
Source: Nat Commun. 2018 May 23;9:2038. doi: 10.1038/s41467-018-04386-3 (PMC5966440; doi:10.1038/s41467-018-04386-3)
Supplement: Supplementary file 1 — Supplementary Information [file 41467_2018_4386_MOESM1_ESM.pdf]

Supplementary Note 1

Among the challenges in studying the role of the excitation energy in free charge formation is ensuring that most carriers originate from charge transfer across the D-A heterojunction and that non-geminate losses prior to extraction are negligible. We screened a wide selection of polymer:fullerene blends searching for systems that satisfied the following constraints: Firstly, prompt non-geminate recombination or fast pseudo-first-order recombination reported in some polymer:fullerene blends must be absent to avoid appreciable free carrier losses prior to the application of the collection bias [1]. Secondly, the efficiency and field dependence of charge generation must be independent of laser fluence in the considered field and intensity range, thereby excluding higher order effects during generation and extraction. Finally, blends had to show an appreciable field dependence of generation, indicating hindered free charge generation. Blends that were not considered further are displayed in Supplementary Table 1.

**blend**	**issues**
PTB7:PCBM	Direct charge generation in too large PCBM domains
PCDTBT:PCBM	Fast initial non-geminate recombination
MEH-PPV:PCBM	Fluence dependent field dependence of charge generation and fast initial non-geminate recombination
2F-PCPDTBT:PCBM	CT- and bulk-emission is too close to determine the CT-state
P3HT:PCBM	No field dependent charge generation

Supplementary Table 1: Overview of other blends tested with TDCF and the reasons why these blends were not considered further in terms of suitability for temperature dependent charge generation measurements.

Supplementary Note 2: Recombination dynamics

Recombination dynamics were measured to ensure that the TDCF data measured at a delay time of 4 ns did not suffer from dispersive recombination or that CT states with a lifetime above ca. 4 ns exist in the sample, which would dissociate during application of the collection bias and contribute to the collected charge. Figure 1a, b plots the charge extracted by TDCF for the PCPDTBT:PCBM blend at room temperature as a function of the delay time between photoexcitation and the onset of the collection bias, ranging between 4 *n*s and several μs (see also Supplementary Note 2). The data show that charges survive approximately 20 *n*s without appreciable recombination at low enough fluence and that the decay follows exactly a second-order recombination process, with the non-geminate recombination coefficient k_2 being independent of the fluence and excitation wavelength. The absence of an initial first order decay rules out that dissociable CT states with a lifetime above *ca.* 4 *n*s exist in the sample, as such states would contribute to the extracted charge at early times. Reducing the temperature causes bimolecular recombination to slow down, as expected (Supplementary Figure 1c). None of the measurements reveal evidence for an appreciable first-order recombination (which would be indicative of the dissociation of long-lived CT states) or dispersive recombination. For the PCPDTBT:ICBA sample, TDCF recombination traces decay faster, but are again consistent with predominant bimolecular free charge recombination (Supplementary Figure 1e). These investigations were complemented by measurements of the decay dynamics of the CT photoluminescence, revealing CT decay times of the order of few nanoseconds (Supplementary Figure 1d and f). These PL transients also show faster decays at higher temperatures, which is expected if dissociation of CT states into free charges competes with their radiative recombination. Note that PL decays recorded by Loi and coworkers on a PCPDTBT:PCBM blend film were virtually unaffected by temperature [2]. In contrast to our measurements those TRPL traces were recorded at around 1100 nm, which is at the low energy side of the CT PL spectrum, and covered only the first 1000 *n*s. Differences between the two sets of measurements might also be due to different degrees of phase separation in the blend layers. In fact, for more phase-separated blends exhibiting pure and intermixed domains, most free charges may be generated from CT states formed at domain boundaries while PL origins mostly from the intermixed domains (the morphology-derived two pool model as proposed by Ref. [3]). Given the very good correspondence of the EL and PL in our PCPDTBT:PCBM devices, and the almost complete lack of a 100 polymer lamellar stacking peak in the corresponding GIWAXS measurements, we propose that the blend is well intermixed. The morphology might have been different for the sample studied by Loi and coworkers (which may also explain why a reverse bias of -2 V had such a small effect on their absolute PL intensity) [4].

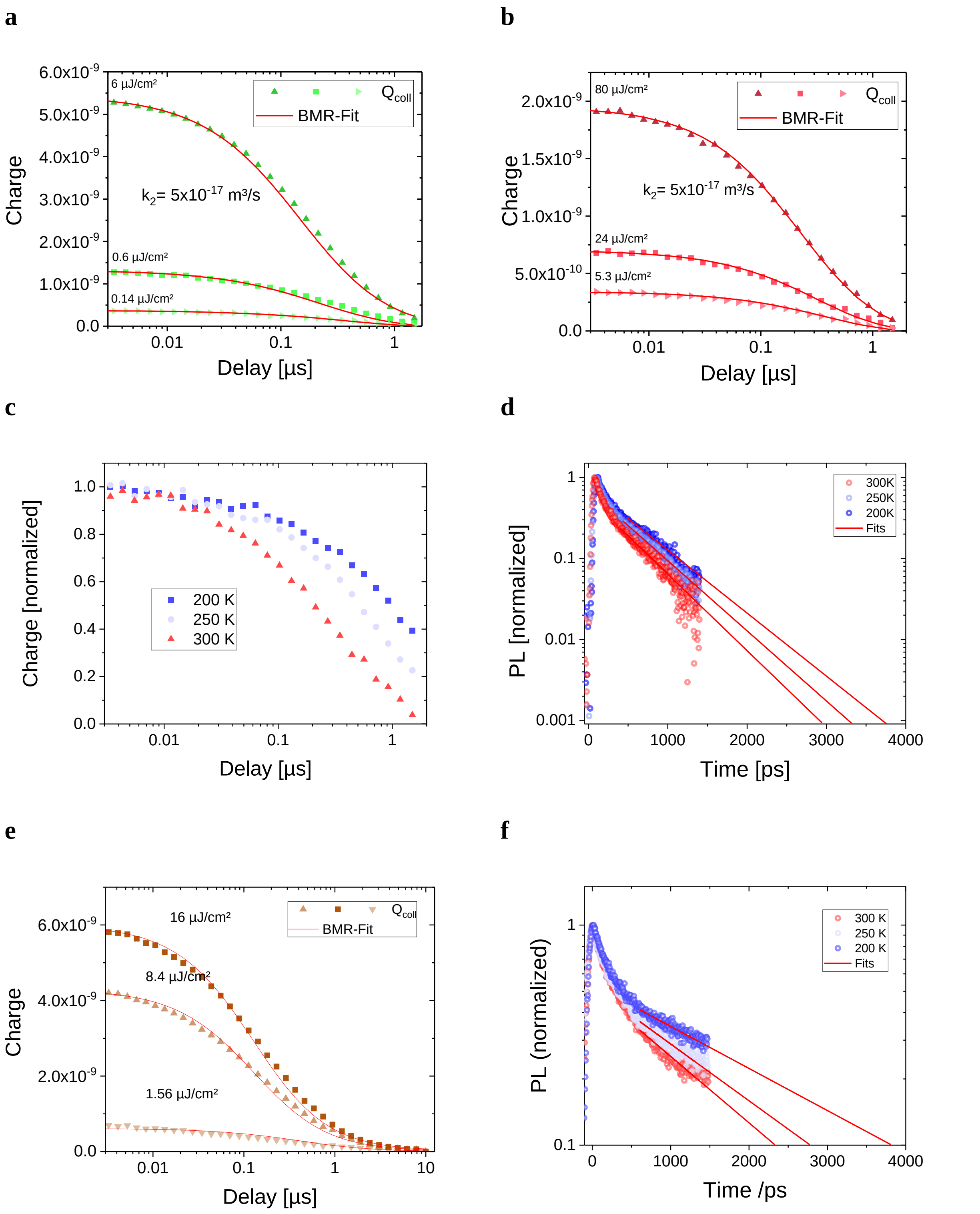

Supplementary Figure 1: Geminate and non-geminate recombination dynamics; Collected charge as a function of the delay time between the laser pulse and the onset of the collection field for the PCPDTBT:PCBM blend, measured with TDCF and excitation at (a) 532 nm and (b) direct CT-excitation at 964 nm. We note, that the beam for direct CT-excitation was focused onto the sample; therefore the fluence stated above is only an approximation. The data were fitted with an iterative fit routine specified in Ref [6] with a single k_2= 5x10^{-17} m³ s^{-1} and a dark charge of Q_{dark}= 1x10^{-10} C. (c) TDCF recombination dynamics for PCPDTBT:PCBM, measured for three different temperatures. The sample had been excited at 964 nm and 6 μJ cm^{-2}. (d) Corresponding time resolved CT-photoluminescence traces for a PCPDTBT:PCBM blend film evaluated in the spectral range from 980 nm to 1180 nm. (e) TDCF recombination dynamics for PCPDTBT:ICBA for three fluences, together with fits to a bimolecular recombination process, (f) time resolved CT-photoluminescence traces for a PCPDTBT:ICBA blend film evaluated from 920 nm to 980 nm.

Supplementary Note 3: Absorbance of the materials in solution, and of neat and blend films

Supplementary Figure 2 compares absorption spectra of the neat materials and the blends in solution and solid state. While the solution spectrum of PCPDTBT is in accordance to fully dissolved polymer chains, the 1F-PCPDTBT solution exhibits contribution of aggregated chains, indicating a stronger tendency of the fluorinated polymer to aggregate already in the solute state (Supplementary Figure 2a), as reported before [5,6]. As a consequence, the absorption of the neat films reveals a more pronounced aggregate peak for the fluorinated polymer (Supplementary Figure 2b and c). The ability of the two polymers to aggregate differently results in a large difference in the absorption of the two polymers in presence of the fullerene (see Supplementary Figure 2d). While the polymer aggregate peak is well maintained in the fluorinated blend, non-aggregated chains dominate the polymer absorption in both PCPDTBT-based blends, with slightly more order in the PCBM-based blend (Supplementary Figure 2d). Notably, the effect of fluorination on the blend absorption is significantly more pronounced than reported earlier by us, which we believe is caused by the higher molecular weight of the 1F-PCPDTBT batch employed here (M_w = 55.000 g mol^{-1} versus 21.500 g mol^{-1} in Ref [6]).

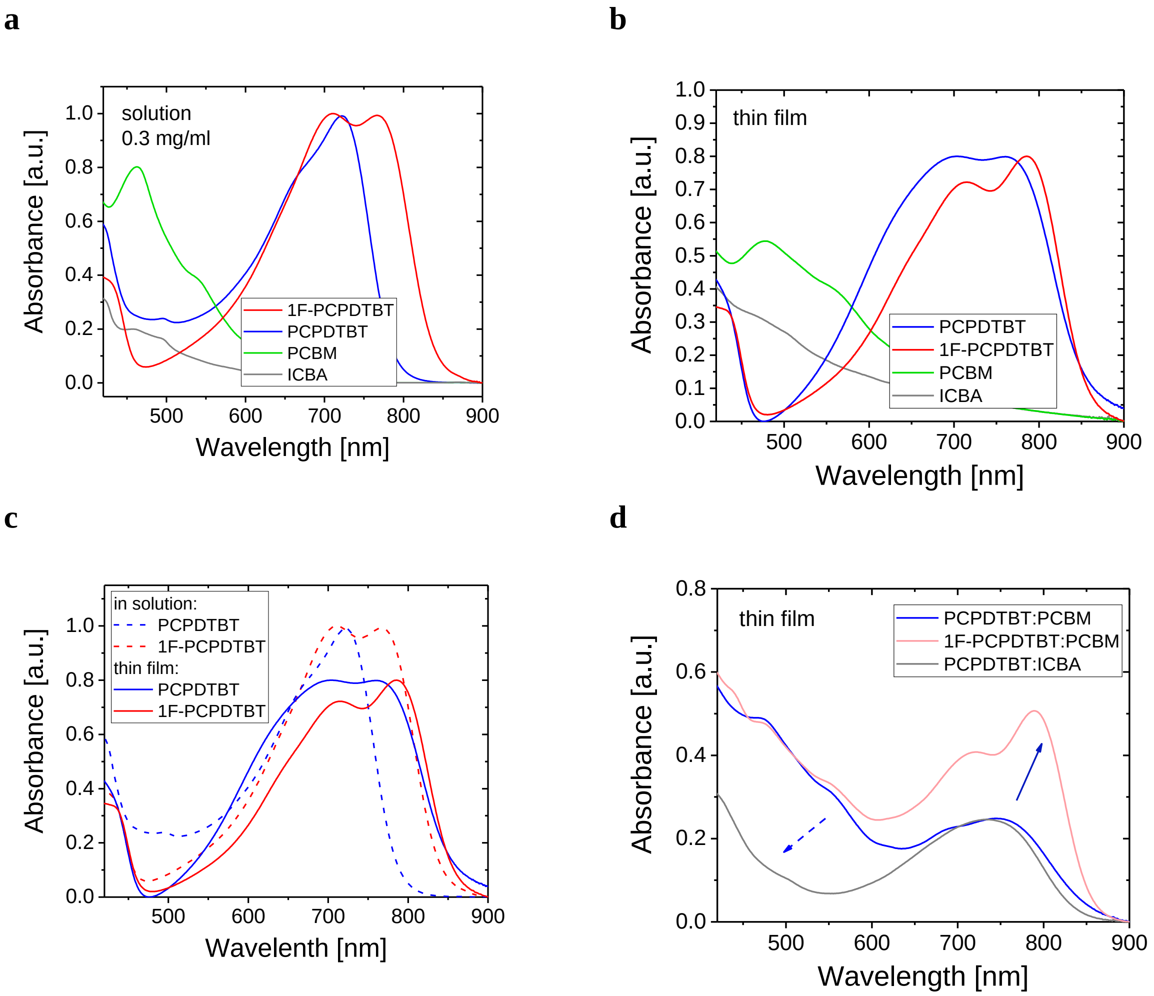

Supplementary Figure 2: Thin Film and in Solution Absorbance (a) Absorbance of all materials used in this work in a 0.3 mg ml^{-1} concentrated solution in Chlorobenzene (1 mm light path length) and (b) in the solid film. (c) Comparison of the polymer absorption in solution and solid state, highlighting the differences in aggregation properties of the two polymers. (d) Blend absorption spectra for 200 nm thick films. Arrows indicate changes in the polymer and fullerene absorption upon fluorination of the polymer (solid blue arrow) or when replacing PCBM by ICBA (dashed blue arrow).

Supplementary Note 4: Peak fits of the GIWAXS profiles of the pure components and the three studied blends

Shown in Supplementary Figure 3 are GIWAXS spectra of neat solid films of all components studied in this work vertically offset for viewing. Blend film peak identification was achieved from direct comparison to scattering from pure components. The thickness of each layer was 110 nm and each spectra were measured under the same conditions as the blends. We note a feature at $Q = 1.4A^{-1}$ for each polymer component corresponding to a PSS layer underneath each polymer film [7].

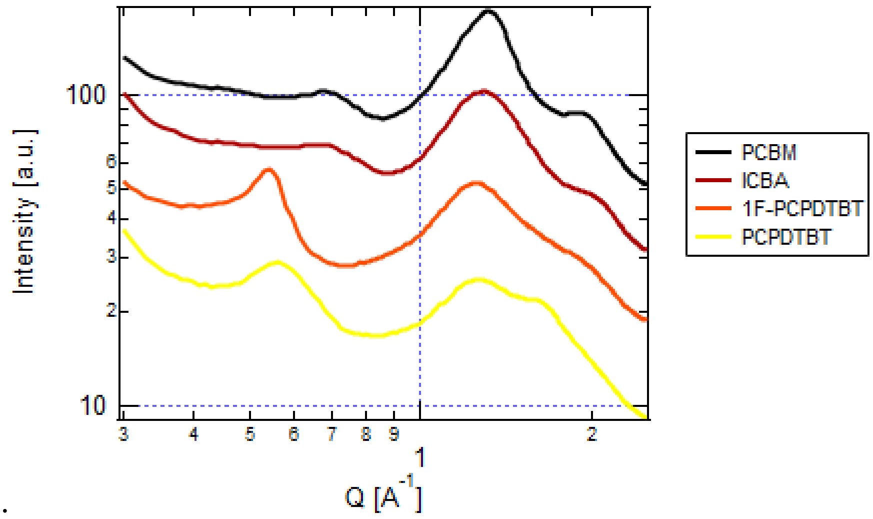

Supplementary Figure 3: GIWAXS of isolated device components found in this study at an incident angle of 2°.

GIWAXS profiles were fit to a series of Lorentzian functions along with linear backgrounds to quantitatively compare polymer/fullerene aggregation. Coherence length is a measure of the average distance over which a periodic structure is established and is calculated for each feature as $2\pi/\text{FWHM}$. All peak fitting is shown with data in Supplementary Figure 4. Features at the lowest Q value were fit independent of other peaks to account for changing backgrounds. The central peak is a convolution of fullerene aggregation and PSS sublayer. We note that when comparing peak widths the PSS layer is constant between different blends, therefore the calculated correlation lengths discussed in the text tracks changes in fullerene aggregation. Parameters deduced from these fits are summarized in Supplementary Table 2

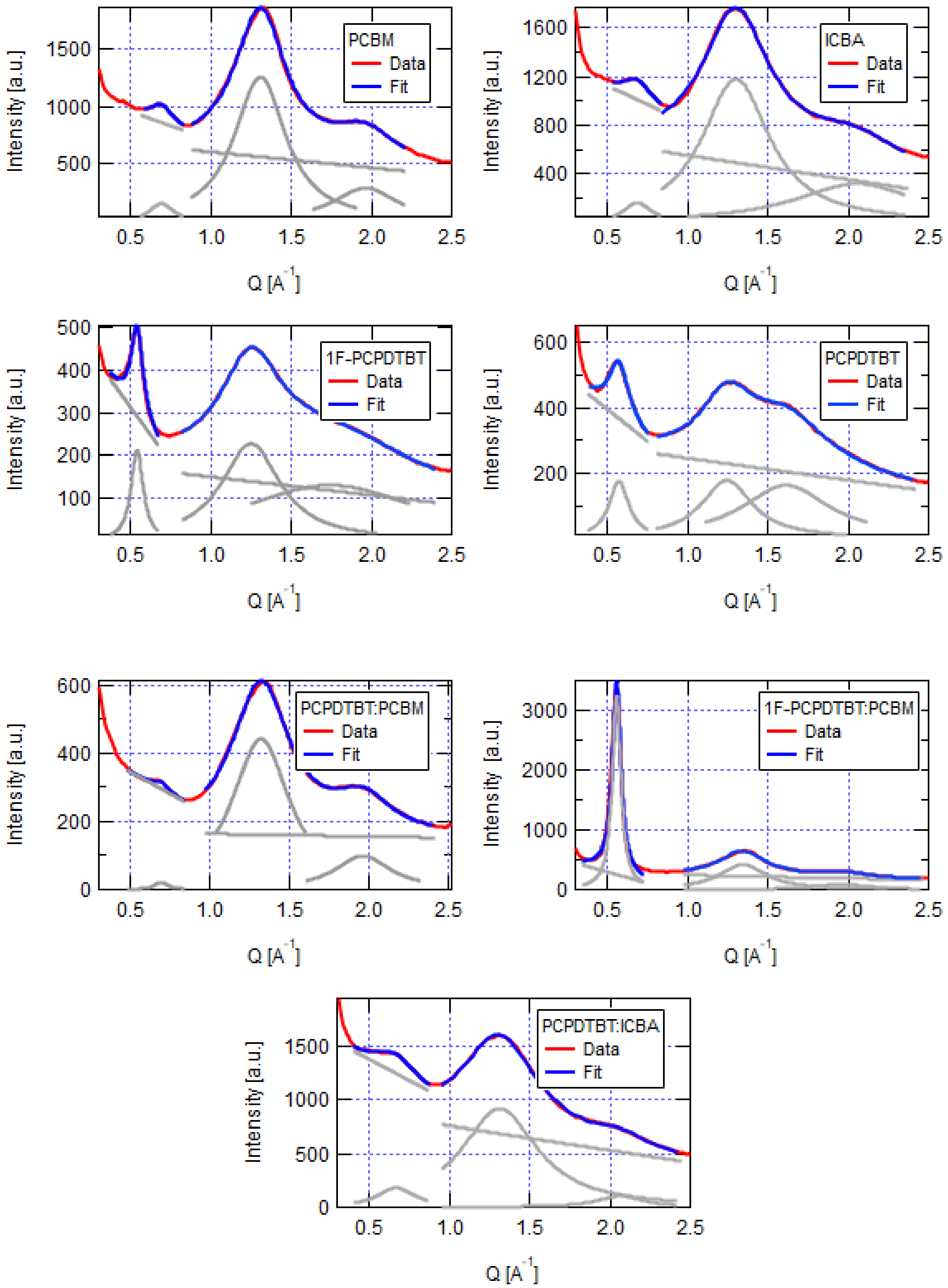

Supplementary Figure 4: Peak fits for all GIWAXS profiles. Shown are the experimental GIWAXS lineouts (red) of all films (both pure and devices) together with corresponding peak fits (blue). eature was fit with a Lorentzian function and a linear background given in grey.

Supplementary Table 2: Compilation of GIWAXS fit results for OPV blends and pure components. Peak identification is in order of lowest to highest values of $\boldsymbol{Q}$ $[\mathbf{A^{-1}}]$ and may not represent the same feature in each sample.

**blend**	Peak 1 Location $[\mathbf{A^{-1}}]$	Peak 2 Location $[\mathbf{A^{-1}}]$	Peak 3 Location $[\mathbf{A^{-1}}]$	Peak 1 FWHM $[\mathbf{\Delta A^{-1}}]$	Peak 2 FWHM $[\mathbf{\Delta A^{-1}}]$	Peak 3 FWHM $[\mathbf{\Delta A^{-1}}]$
PCPDTBT:PCBM	0.688(2)	1.3210(9)	1.963(3)	0.09(5)	0.436(5)	0.42(2)
PCPDTBT:ICBA	0.666(3)	1.312(1)	2.065(5)	0.3(1)	0.577(8)	0.34(3)
1F-PCPDTBT:PCBM	0.556(2)	1.3437(7)	1.963(3)	0.06(1)	0.353(3)	0.37(2)
PCBM	0.691(1)	1.3056(8)	1.961(7)	0.17	0.385(3)	0.49(4)
ICBA	0.685(1)	1.300(1)	2.05(3)	0.21	0.511(7)	0.9(1)
1F-PCPDTBT	0.540(1)	1.248(1)	1.74(1)	0.08	0.446(6)	1.39(8)
PCPDTBT	0.5691(1)	1.244(2)	1.612(5)	0.15	0.387(8)	0.66(1)

Supplementary Note 5: Surface Topography

Supplementary Figure 5 displays the topology and phase images of the three blends of interest, taken by AFM in tapping mode. In accordance to the morphology data described above, the PCPDTBT:ICBA blend reveals a rather featureless surface while the surface of the 1F-PCPDTBT:PCBM exhibits fiber-like structures which we attribute to polymer aggregates.

PCPDTBT:PCBM	PCPDTBT:ICBA	1F-PCPDTBT:PCBM
19 nm^2	10 nm^2	25 nm^2
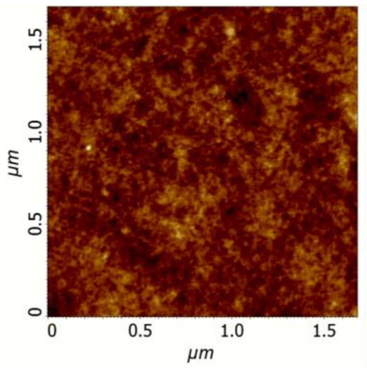	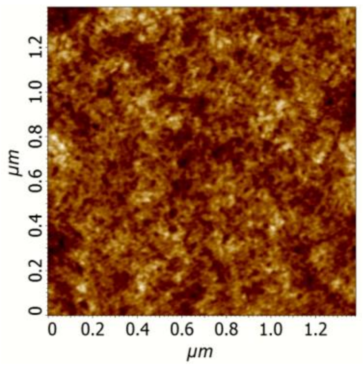	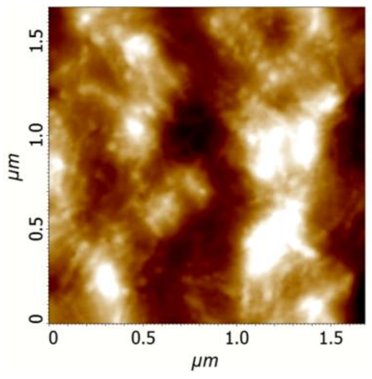
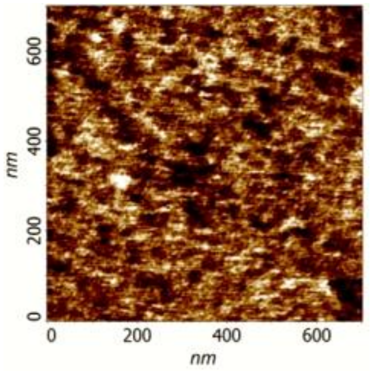	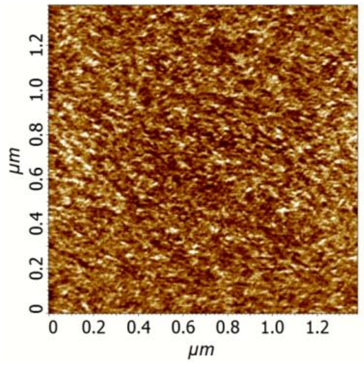	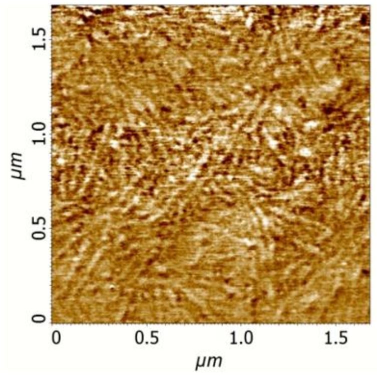

Supplementary Figure 5: AFM topology (upper) and phase (lower) pictures of all blends measured on a 30x30 µm^2 area in the AFM tapping mode phase images. The penetration depth of the tip is typically 6 nm, which results in a great contrast ratio of the surface.

Supplementary Note 6: Excitation Conditions

Special care was taken to ensure that only the selected wavelength is incident on the device We used laserline filters with a FWHM of 10 nm for the fundamental wavelength of 1064 nm and 800 nm, long-pass filters for direct CT excitation to cut out residual higher energy photons, and a 355-650 nm bandpass filter for all other excitation wavelengths. The measured FWHM of the 1064 nm and 532 nm pulses is only 6 nm, which is important for selective excitation. The pulse width is 3.8 *n*s, long enough to reduce the peak power from 761 kW cm^{-2} (typical femtosecond laser system) to 9 kW cm^{-2}, which makes non-linear parametric processes negligible. For all other wavelengths, we used femtosecond pulses that are spectrally broader (FWHM of 20 nm). Selectivity was ensured with the same filters.

Supplementary Note 7: Charge generation as function of temperature and internal electric field

Field and temperature dependence of charge generation in a 150 nm thick PCPDTBT:PCBM blend for an excitation wavelength of 800 nm are shown in Supplementary Figure 6. The internal electric field was calculated from the external bias for a flat band potential of 0.8 V. The dependence of the collected charge as function of bias plotted in a log-log fashion displays a linear increase for higher fields, which passes into a nearly flat plateau when approaching open circuit conditions.

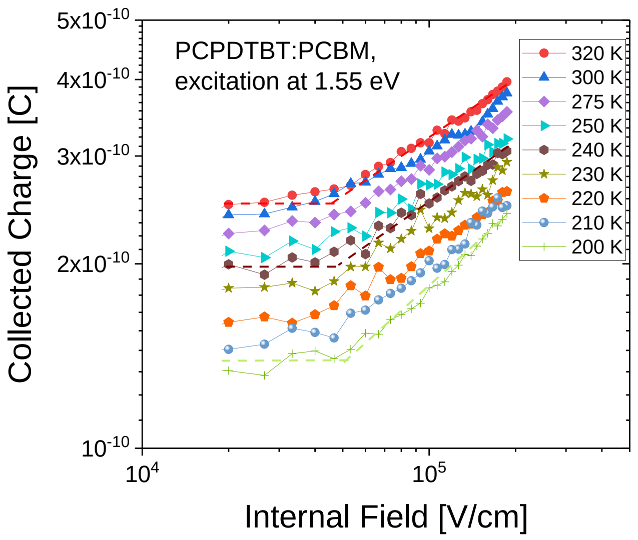

Supplementary Figure 6: Temperature and electric field dependent charge generation in the 110 nm thick PCPDTBT:PCBM blend for an excitation wavelength of 800 nm. A flat band potential of +0.8 V was used in the calculation of the internal electric field.

Supplementary Note 8: Field dependence of charge generation

In Supplementary Figure 7 field dependent TDCF charge generation data are summarized for the highest (300 and / or 340 K) and lowest (200 K) applied temperatures for all relevant excitation photon energies including direct CT and CT-emission-maximum excitation. The active layer thickness is 110 nm. All data were normalized to the corresponding charge at a reverse bias of -2 V to show possibly diverging slopes in the field dependence with varying temperature or photon energy. For PCPDTBT:PCBM (Supplementary Figure 7a), 1F-PCPDTBT:PCBM (Supplementary Figure 7b) and PCPDTBT:ICBA (Supplementary Figure 7c) only a very small change in the slope caused mainly by temperature is observable, meaning that charge generation is essentially independent of excitation energy.

a

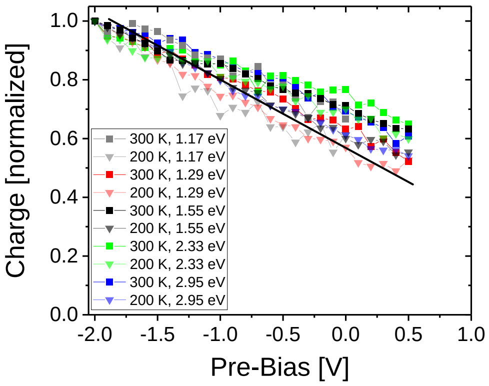

b

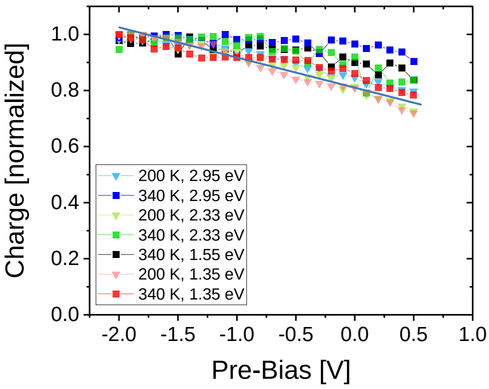

c

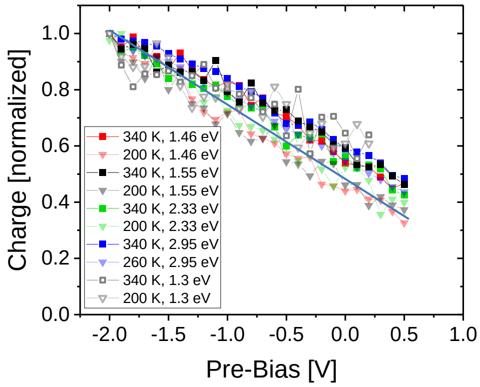

Supplementary Figure 7 Field and temperature dependent charge generation for PCPDTBT:PCBM (**a**), 1F-PCPDTBT (**b**) and PCPDTBT:ICBA (**c**). Solid black lines indicate the field dependence under direct CT excitation and a temperature of 200 K (triangles).

Supplementary Note 9: Electroluminescence spectra as function of bias

Electroluminescence spectra were recorded as function of bias to test whether states of different nature contribute to the EL signal. This seems to be clearly the case for the 1F-PCPDTBT:PCBM blend, where we assign the high energy feature to emission from well-aggregated 1F-PCPDTBT chains (Supplementary Figure 8a). In contrast, the shape of the PCPDTBT:PCBM blend is fully independent of bias (Supplementary Figure 8b). We conclude that emission from aggregated polymer chains is either weak (because of the low degree of aggregation in this blend) or that singlet states and CT states thermalize through rapid singlet exciton splitting and reformation.

a **b**

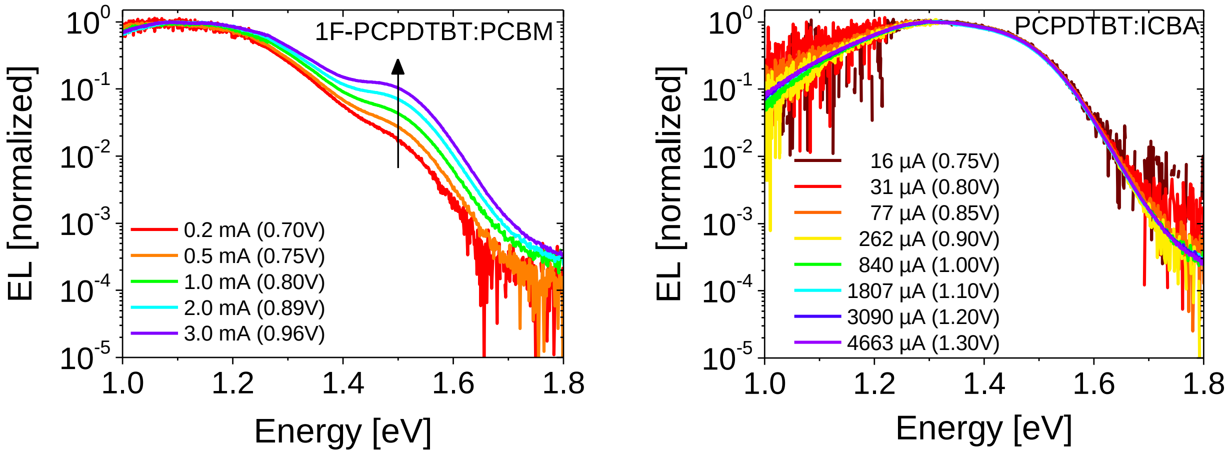

Supplementary Figure 8: Room temperature bias-dependent electroluminescence spectra of devices made from 1F-PCPDTBT (**a**) and PCPDTBT:ICBA (**b**). Spectra were normalized to their maximum.

Supplementary Note 10: Calculating Photoluminescence (PL) Quenching Efficiency

If the absolute PL at zero internal field is not accessible (or dominated by EL), it is also convenient to refer the efficiency of PL quenching to a different voltage.

In a simple rate model the voltage dependent PL intensity can be written as:

$$\mathrm{PL}(V) = A\frac{k_\mathrm{r}}{k_\mathrm{d}(V)+k_\mathrm{r}+k_\mathrm{nr}} \tag{1}$$

where the fraction on the right side of Supplementary Equation 1 in the absolute PL quantum efficiency and A is an unknown prefactor taking into account all other factors determining the PL intensity.

The CT dissociation efficiency is given by:

$$\eta_\mathrm{d}(V) = \frac{k_\mathrm{d}(V)}{k_\mathrm{d}(V)+k_\mathrm{r}+k_\mathrm{nr}} \tag{2}$$

where k_r is the radiative-, k_nr the non-radiative-recombination- and $k_\mathrm{d}(V)$ the dissociation-rate. We assume, that only k_d is voltage dependent. Now, we define the PLQE quenching with reference to an arbitrary voltage V_0, with $\mathrm{PL}(V) < \mathrm{PL}(V_0)$:

$$\mathrm{PLQE}(V) \stackrel{\mathrm{def}}{=} 1 - \frac{\mathrm{PL}(V)}{\mathrm{PL}(V_0)} \tag{3}$$

We now show that the PLQE(V) referenced to V_0 is given by Equation 1 in the main text:

$$\mathrm{PLQE(V)} = \frac{\eta_\mathrm{d}(V)-\eta_\mathrm{d}(V_0)}{1-\eta_\mathrm{d}(V_0)} \tag{4}$$

For this, we rewrite Supplementary Equation 4:

$$\mathrm{PLQE(V)} = \eta_\mathrm{d}(V) - \eta_\mathrm{d}(V_0)(1 - \mathrm{PLQE(V)}) \tag{5}$$

Now the definition for $\eta_\mathrm{d}(V)$, Supplementary Equation 4 and 5 leads to:

$$\mathrm{PLQE(V)} = \frac{k_\mathrm{d}(V)}{k_\mathrm{d}(V)+k_\mathrm{r}+k_\mathrm{nr}} - \frac{k_\mathrm{d}(V_0)}{k_\mathrm{d}(V_0)+k_\mathrm{r}+k_\mathrm{nr}}\frac{\mathrm{PL}(V)}{\mathrm{PL}(V_0)} \tag{6}$$

Finally, with Supplementary Equation 1 and resorting, the right hand side can be rewritten as:

$$1 - \frac{k_\mathrm{d}(V_0) + k_\mathrm{r} + k_\mathrm{nr}}{k_\mathrm{d}(V) + k_\mathrm{r} + k_\mathrm{nr}} = 1 - \frac{\mathrm{PL}(V)}{\mathrm{PL}(V_0)}$$

which exactly is PLQE(V) as defined above. These equations also show that it is not necessary to measure absolute PL efficiencies in an integrating sphere, as only the relative PL intensities enter Supplementary Equation 1.

Supplementary Note 11: Determination of the CT splitting efficiency from TDCF measurements

Evaluation of Equation 1 requires knowledge of the absolute efficiency of field-induced CT splitting. Because of the onset of a considerable dark current for reverse bias for the 1.1 mm² device areas, we limited the voltage range for TDCF measurements to -2V for the PCPDTBT:PCBM blend and to -4 V for the blend with ICBA. The absolute CT splitting efficiency $\eta_{\mathrm{diss}}(E)$ was then obtained by scaling the bias-dependent TDCF data to a properly normalized photocurrent characteristic from large 16 mm² devices on the same substrate, assuming that the saturation of the photocurrent at large reverse bias corresponds to η_{diss} equaling one. The so-obtained dependence of η_{diss} on external voltage is plotted in Supplementary Figure 9, with a well-discernible splitting efficiency at the reference voltage of $\eta_{\mathrm{diss}}(0.5\ \mathrm{V})$ equaling 0.46±0.01 and 0.36±0.01 for PCPDTBT:PCBM and PCPDTBT: ICBA, respectively.

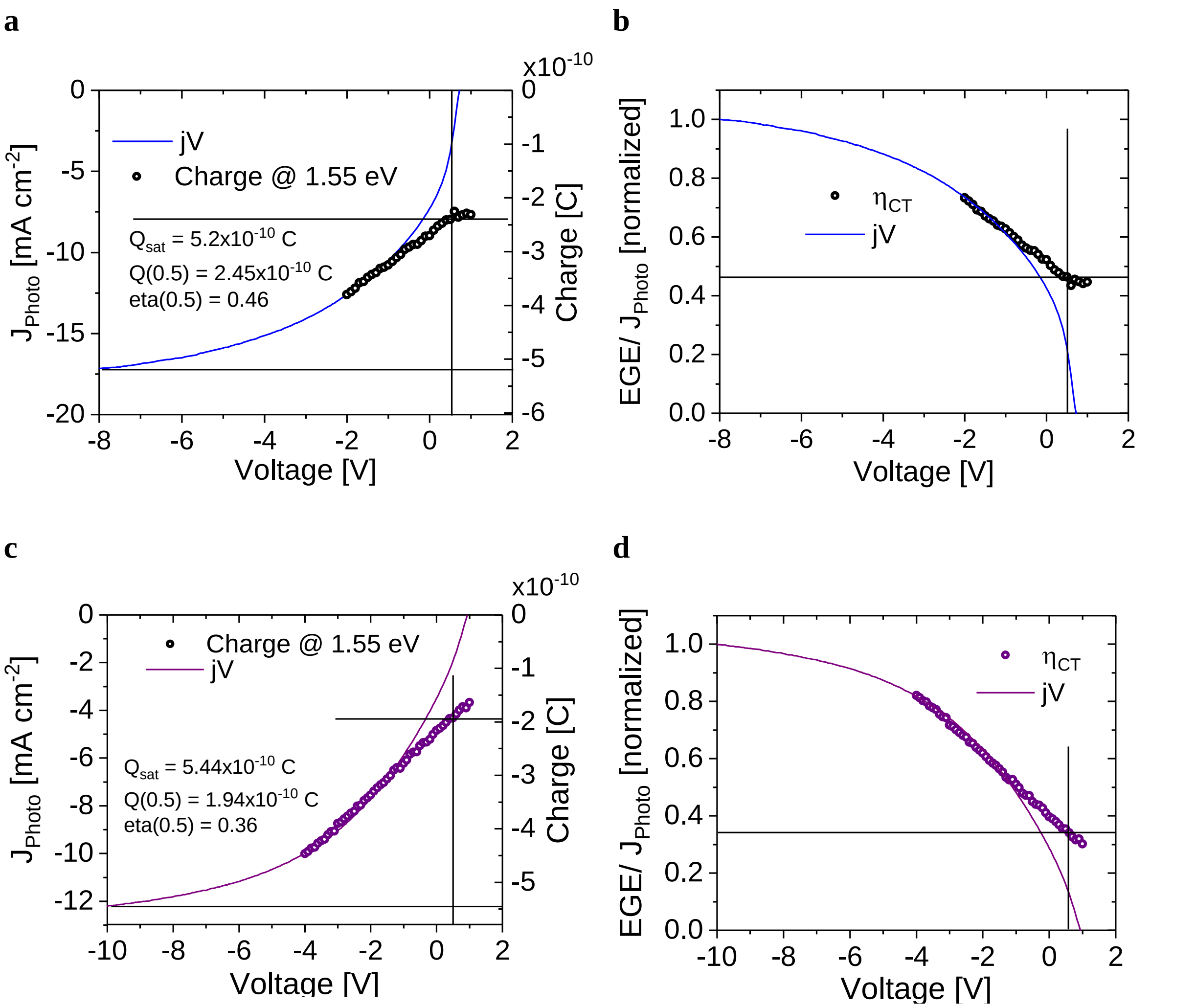

Supplementary Figure 9: Photogenerated Charge versus Photocurrent to obtain the dissociation efficiency as function of bias. Shown by the solid line is the photocurrent (light current minus dark current, right column normalized of a) & b) PCPDTBT:PCBM and c) & d) PCPDTBT:ICBA solar cell under simulated one sun illumination at room temperature. The field-dependence of the extracted charge from TDCF experiments at 300 K is then scaled to give the best agreement to photocurrent characteristics. Data are shown for excitation with a photon energy of 1.55 eV, at the polymer bandedge. As pointed out in the main text, there is no discernible dependence of the field dependent charge generation on excitation energy, meaning that the formation of the CT state from highly-excited excitons is not affected by the electric field. The scaled TDCF data therefore yield the field-dependent efficiency for CT splitting, $\eta_{\mathrm{diss}}(V)$. The well-discernible splitting efficiency at the reference voltage $\eta_{\mathrm{diss}}(0.5\ V)$ is directly shown in the graphs.

Supplementary Note 12: Photoluminescence and Electroluminescence of 1F-PCPDTBT neat Films and Blends with PCBM

Although the EL of the 1F-PCPDTBT:PCBM blend has a well discernible CT-emission spectra energetically below the PL emission of the neat 1F-PCPDTBT (orange versus brown line in Supplementary Figure 10), the PL of the blend is mainly dominated by the PL from aggregated polymer chains. This can be also seen in distinct identifiable peaks in absorbance (see Supplementary Figure 2) and emission (Supplementary Figure 10, black line) in combination with the GIWAXS results (see Supplementary Figure 3 and 4) pointing to the very strong aggregation of this particular system.

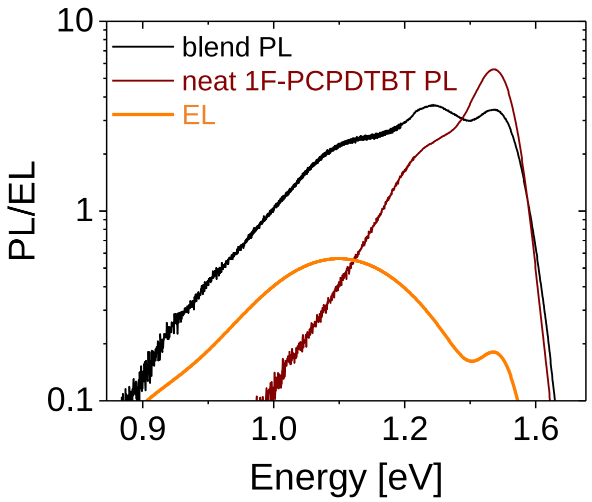

Supplementary Figure 10: Photoluminescence in comparison to Electroluminescence. PL for a 1F-PCPDTBT:PCBM blend (black) and the neat polymer PL (dark red) in comparison to the EL (orange).

Supplementary References

1 Kurpiers, J. & Neher, D. Dispersive Non-Geminate Recombination in an Amorphous Polymer:Fullerene Blend. *Sci. Rep.* **6,** 26832 (2016).

2 Jarzab, D. *et al.* Low-Temperature Behaviour of Charge Transfer Excitons in Narrow-Bandgap Polymer-Based Bulk Heterojunctions. *Adv. Energy Mater.* **1,** 604–609 (2011).

3 Tvingstedt, K., Vandewal, K., Zhang, F. & Inganäs, O. On the Dissociation Efficiency of Charge Transfer Excitons and Frenkel Excitons in Organic Solar Cells: A Luminescence Quenching Study. *J. Phys. Chem. C* **114,** 21824–21832 (2010).

4 Jarzab, D. *et al.* Low-Temperature Behaviour of Charge Transfer Excitons in Narrow-Bandgap Polymer-Based Bulk Heterojunctions. *Adv. Energy Mater.* **1,** 604–609 (2011).

5 Zhang, Y., Zou, J., Cheuh, C. C., Yip, H. L. & Jen, A. K. Y. Significant improved performance of photovoltaic cells made from a partially fluorinated cyclopentadithiophene/benzothiadiazole conjugated polymer. *Macromolecules* **45,** 5427–5435 (2012).

6 Albrecht, S. *et al.* Fluorinated Copolymer PCPDTBT with Enhanced Open-Circuit Voltage and Reduced Recombination for Highly Efficient Polymer Solar Cells. *J. Am. Chem. Soc.* **134,** 14932 (2012).

7 Rivnay, J. *et al.* Structural control of mixed ionic and electronic transport in conducting polymers. *Nat. Commun.* **7,** 11287 (2016).
